# Supplementary material for: Lipid-Associated GWAS Loci Predict Antiatherogenic Effects of Rosuvastatin in Patients with Coronary Artery Disease
Source: Genes (Basel). 2023 Jun 13;14(6):1259. doi: 10.3390/genes14061259 (PMC10298211; doi:10.3390/genes14061259)
Supplement: Supplementary file 1 [file genes-14-01259-s001.zip › genes-2348537-supplementary.pdf]

Supplementary Table S1. Change in lipid and carotid intima-media thickness values during rosuvastatin therapy.

Parameter	Baseline value	On-treatment value (6 months of therapy)	On-treatment value (12 months of therapy)
TC, mmol/l	5.28 (4.60; 6.06)	3.34 (3.05; 3.60) *	3.31 (3.10; 3.57) *
LDL-C, mmol/l	3.26 (2.70; 4.08)	1.62 (1.35; 1.74) *	1.65 (1.45; 1.75) *
HDL-C, mmol/l	1.06 (0.97; 1.29)	1.08 (0.95; 1.21) ****	1.12 (0.97; 1.24) ****
TG, mmol/l	1.71 (1.22; 2.37)	1.25 (1.07; 1.84) *	1.28 (1.01; 1.62) *
CIMT, maximum, mm	0.80 (0.60; 1.00)	0.70 (0.50; 0.80) *	0.60 (0.50; 0.80) *
CIMT, mean, mm	0.70 (0.55; 0.85)	0.65 (0.53; 0.80) ***	0.60 (0.53; 0.75) **

* p-value $< 1\times10^{6}$ for comparison of baseline value with on-treatment value. ** p-value $< 1\times10^{5}$ for comparison of baseline value with on-treatment value. *** p-value = 0.025 for comparison of baseline value with on-treatment value. **** p-value > 0.1 for comparison of baseline value with on-treatment value.
